# A Reasonable Course to Pursue in the Treatment of Pulpless Teeth

**Published:** 1917-11

**Authors:** Otto U. King


### A REASONABLE COURSE TO PURSUE IN THE TREATMENT OF PULPLESS TEETH

BY OTTO U. KING, D.D.S.

The dental profession has long sought for a safe and reliable method for cleansing, sterilizing and filling root canals. That the essential necessities for success have been little understood is evidenced by the widely diversed methods followed by men in different parts of the country, and also because of the enormous percentage of failures revealed by the X-ray. The facts recently developed by the research commission showing the universality of infections of root ends of pulpless teeth are unquestioned and many dentists and physicians have accepted them as final and have adopted the forceps as a cure for infections of all pulpless teeth. This is indeed unfortunate and unwarranted. The assumption that infections occurring about the ends of pulpless teeth are blood borne from distant parts of the body is also without foundation; first, because routine blood cultures in a large number of cases of individuals who have had dental abscess infection about the ends of pulpless teeth have failed to reveal bacteria in the blood stream; second, because the bacteria found in the chronic dental granuloma is by all cultural methods proven to be the same type of bacteria as that which is found on the surfaces of teeth, and all the facts in the case point to the pulp canal and pyorrhea pocket as the ports of entry through which these bacteria enter and affect lodgment in the apical areas. One of the most reliable pieces of work done to solve the problem of the channel of root infection is that of B. Mayrhofer of the University of Innsbruck, under the title of "Klinisch-bakteriologische Untersuchungen zur Pathologie und Therapie der faulen Zahnpulpa. Jena, 1909, 1910. Wiener klinische Wochenschrift, 21, 615." Mayrhofer found that the constant inhabitant of the deep portion of the root canal to be the streptococcus. He also found that teeth might be treated by the Buckley method, formalin cresol, or Schreir's sodium and potassium and apparent sterility would be gained, but if the canals were filled by the ordinary methods and with the ordinary type of root canal filling material, that in a course of a very few weeks removal of the root canal filling and culture would reveal growth in the apices of the previously filled roots. He therefore made the deduction that sterilization of the extreme root ends had not been accomplished and that after a period of incubation had been allowed to elapse, the bacteria became numerous enough to yield positive culture. He therefore adopted the method of filling the root ends with Perubalsam, an adhesive varnish, which he believed penetrated the bases of the tubules, and efficiently prevented the further growth of any streptococci left in the root tips, thereby greatly increasing the percentage of his successes in filling the roots of pulpless teeth.

An examination of several hundred root tips obtained and examined under aseptic precautions by Hartzell and Henrici confirm Mayrhofer's view that the infectious microorganism causing dental abscesses is resident in the root end itself. Therefore, the problem of successfully sterilizing and filling root canals is to be solved by adopting methods that effectively destroy the bacteria in the tips of the canal, as well as in the pulp chamber. That this can be done has been amply proven by the work of Callahan, Rhein, Best and others and it is therefore indeed unfortunate that the *Dental Cosmos* in its editorial columns should give utterance to the view that the old and sloppy method of root canal filling with cotton should have any standing in court. It is the belief of the editors of this journal that root canal operations should be conducted with exactly the same aseptic precautions in the way of clean instruments clean materials, clean hands and sterility of the canals themselves that are expected of the general surgeon in opening a brain or removing an appendix, and that where the dentist adopts these methods from start to finish in his root canal work, teeth so treated will remain in the jaws and do service with entire safety to the patient. The accumulating evidence of the past two years shows conclusively that it is possible to open the root tips of canals, sterilize and fill them, and even permit a quantity of chlora percha to extrude through the root end without permanent damage to the tissues beyond. The evidence thus far collected shows that the temporary damage wrought by the use of powerful antiseptics or the extrusion of root canal filling material when sterile is rapidly repaired without permanent damage to the tissues, and the teeth so treated remain in a safe and healthy condition. The rapidly increasing number of radiographic histories of abscessed teeth that have been sterilized and treated by the Callahan method with subsequent amputation of the root ends shows conclusively that if the root itself be sterile, no matter what the trauma at the root end by operation has been, new bone is built into the area vacated by removal of the root tip and diseased tissue. The Callahan method is an improvement on the Mayrhofer method in that it gives us a material which is very sticky and at the same time intensely penetrating, enabling it to flow into and seal the bases of tubules which have been emptied of fibriles by chemical methods, thus preventing the possibility of procreation of streptococci which may have escaped the action of chemical agents employed to sterilize the canals. Callahan's formula, it will be remembered, is thirty-two grains of the finest resin to the ounce of chloroform,* the canal being filled with the chloroform resin mixture with a hard gutta-percha point thrust into it, churning it up and down until the guttapercha is dissolved in the canal, after which additional points are driven firmly home. All such canals should have the pulp chamber third filled with the most impervious cement obtainable to effectually prevent bacteria gaining entrance to the canal from the pulp chamber.

Hartzell and Henrici have collected a large series of radiographic records of teeth which were treated as follows:

*First.* The dead pulps were removed with barbed broaches and dioxogen.

*Second.* The pulp canals were treated by churning into them Schreier's sodium and potassium in order to sterlize and dissolve the fatty material.

*Third.* The root canals were washed out with alcohol in order to free them from the soapy sodium potassium hydrate.

*Fourth.* The canals were flushed with C. P. sulphuric acid.

*Fifth.* The canals were dried out with dry cotton.

*Sixth.* The canals were filled by the Callahan method.

*Seventh*, was the process of apecoectomy.

The development of bone follows, providing the root is sterile and the diseased tissue is all removed.—*Journal N. D. A.*

---

* To this solution, Conzett adds 10 grams of aristol to cause the filling to cast an X-ray shadow.

